# P-1359. A Systematic Review of Prevalence and Epidemiological Characteristics of Carbapenemase-producing Enterobacterales (CPE) in the United Kingdom

**DOI:** 10.1093/ofid/ofaf695.1546

**Published:** 2026-01-11

**Authors:** Uzair Akbar Ali, Tranprit Saluja

**Affiliations:** Univeristy Hospitals Birmingham NHS Trust, Bkirmingham, England, United Kingdom; Sandwell and West Birmingham Hospitals NHS Trust, Bkirmingham, England, United Kingdom

## Abstract

**Background:**

The prevalence of Carbapenemase-producing Enterobacterales (CPE) was low in the United Kingdom before the mid-2000s. CPE-associated infections are challenging to treat, resulting in an increased risk of treatment failure, higher treatment costs, morbidity, and mortality rates. There is a global rise in CPE prevalence, particularly in healthcare settings. Surveillance of CPE is essential for infection control measures and to prevent outbreaks.

Screening of studies included in the review

**Figure 1.**
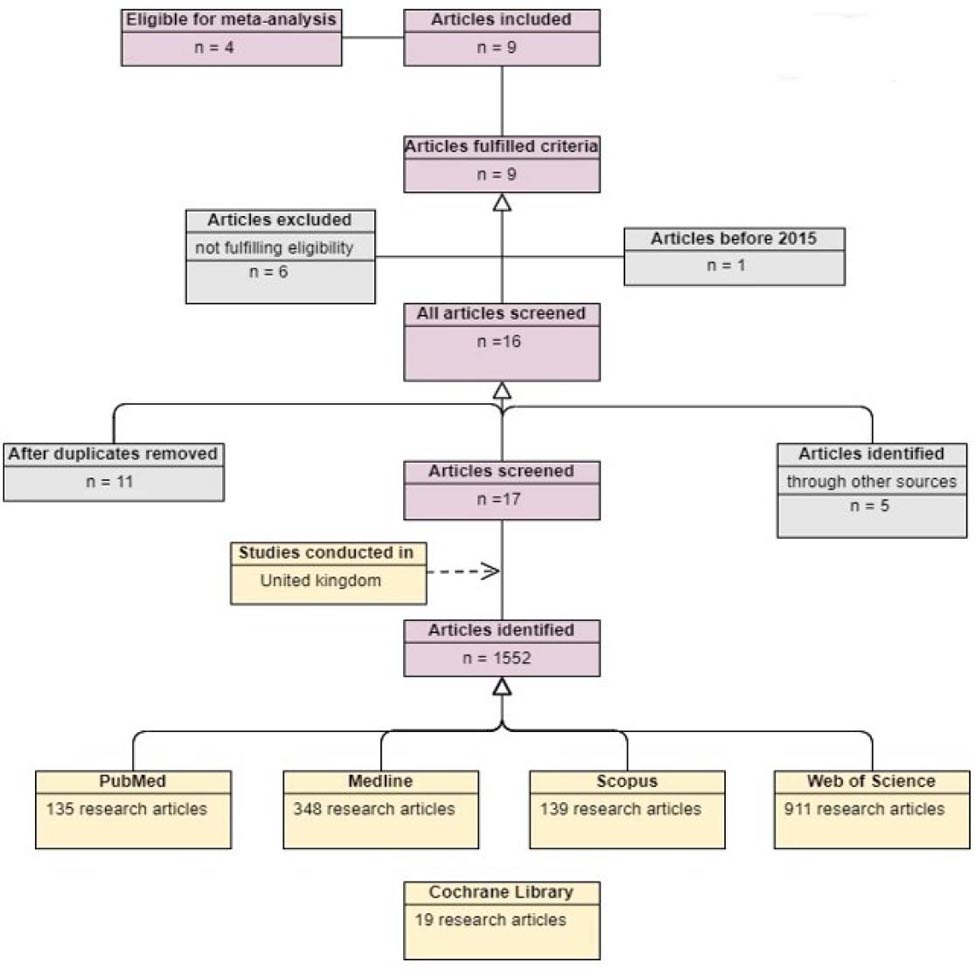
PRISMA flowchart for papers screened for the prevalence and epidemiology of CPE.

**Figure 2.**
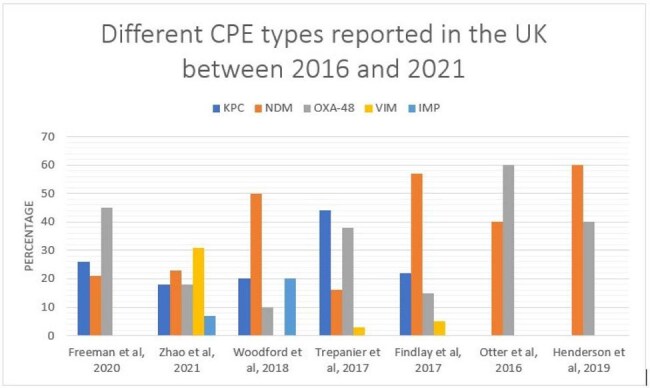
Prevalence of CPE strains in the UK during the period 2003-2019 in seven different studies.

**Methods:**

The study aimed to estimate CPE prevalence in the UK and to determine epidemiological characteristics. A literature search was conducted on PubMed, Medline, Scopus, Web of Science, and the Cochrane Library. The investigation adhered to the Preferred Reporting Items for Systematic and Meta-Analysis (PRISMA) guidelines. Relevant data were extracted and analysed using statistical tools. A meta-analysis of these risk factors was performed.

**Figure 3.**
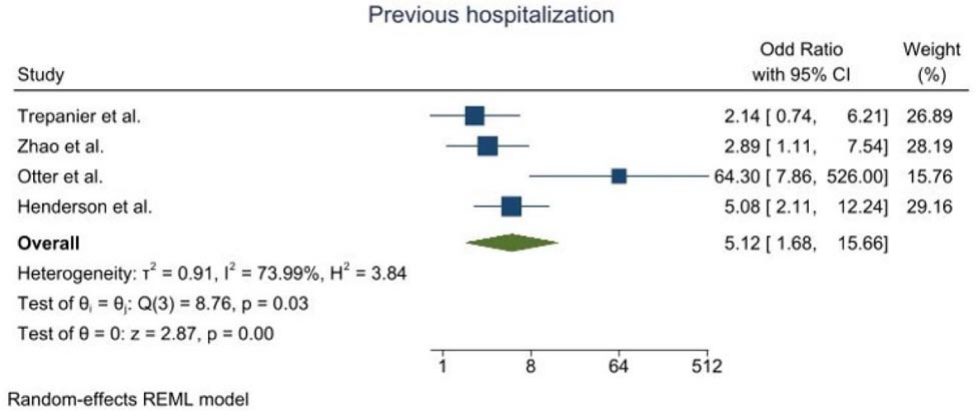
Forest plot for the previous hospitalization as a risk factor of CPE acquisition

**Figure 4.**
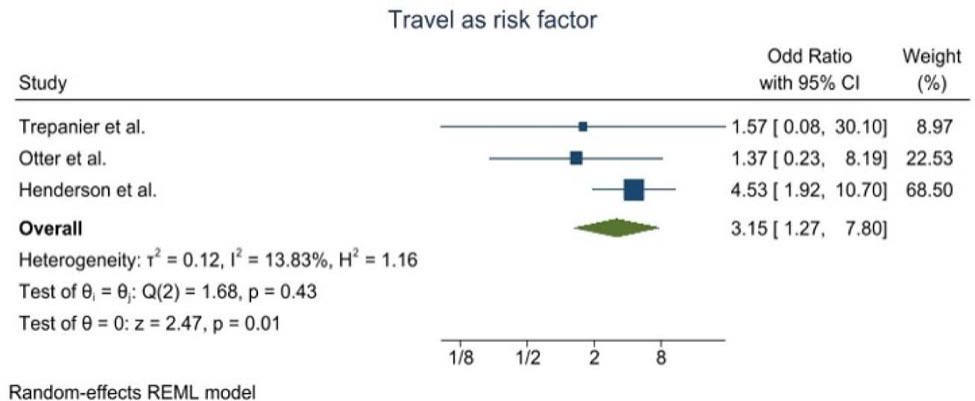
Forest plot for the history of recent travel as a risk factor for CPE acquisition

**Results:**

A total of 1,552 articles were identified, after which they underwent further screening. The review included nine studies that detailed the CPE prevalence in the UK. Overall, CPE prevalence ranged from 0.1% to 3.8%, varying by location. A geographical difference was noted in the distribution of carbapenem resistance genes, with KPC being the dominant type in Manchester, whereas NDM and OXA-48 were primarily found in the West Midlands and London. In Scotland, VIM and NDM were identified as the predominant types. The meta-analysis of risk factors revealed varying degrees of heterogeneity. Notably, previous hospitalization as a risk factor exhibited greater variability, showing an overall heterogeneity of 3.84, compared to travel, which had a heterogeneity value of 1.16.

**Conclusion:**

The review indicated an increase in CPE prevalence across healthcare settings in the UK, showing varied prevalence and resistant genes across regions. Recent hospitalizations and international travel are identified as risk factors for acquiring CPE. These study findings can help investigate the reasons behind the epidemiological spread of CPE, focusing on specific carbapenemase families in different UK regions and the associated risk factors for transmission or localized outbreaks. This could enhance existing Infection Prevention and Control (IPC) practices and CPE screening policies in the UK.

**Disclosures:**

All Authors: No reported disclosures

